# School health services and its practice among public and private primary schools in Western Nigeria

**DOI:** 10.1186/s13104-016-2006-6

**Published:** 2016-04-06

**Authors:** Olugbenga Temitope Kuponiyi, Olorunfemi Emmanuel Amoran, Opeyemi Temitola Kuponiyi

**Affiliations:** Department of Community Medicine and Primary Care, Olabisi Onabanjo University Teaching Hospital, Sagamu, Nigeria; Department of Paediatrics, College of Health Sciences, Olabisi Onabanjo University Teaching Hospital, Sagamu, Nigeria

**Keywords:** School health, Services, Practice, Primary schools, Nigeria

## Abstract

**Background:**

Globally the number of children reaching school age is estimated to be 1.2 billion children (18 % of the world’s population) and rising. This study was therefore designed to determine the school health services available and its practices in primary schools in Ogun state, Western Nigeria.

**Methods:**

The study was a comparative cross-sectional survey of private and public primary schools in Ogun state using a multi-stage sampling technique. Participants were interviewed using a structured, interviewer administered questionnaire and a checklist. Data collected was analyzed using the SPSS version 15.0.

**Results:**

A total of 360 head teachers served as respondents for the study with the overall mean age of 45.7 ± 9.9 years. More than three quarters of the respondents in both groups could not correctly define the school health programme. There were no health personnel or a trained first aider in 86 (47.8 %) public and 110 (61.1 %) private schools but a nurse/midwife was present in 57 (31.7 %) and 27 (15.0 %) public and private schools. (χ^2^ = 17.122, P = 0.002). In about 95 % of the schools, the teacher carried out routine inspection of the pupils while periodic medical examination for staff and pupils was carried out in only 13 (7.2 %) public and 31 (17.2 %) private schools (χ^2^ = 8.398, P = 0.004). A sick bay/clinic was present in 26 (14.4 %) and 67 (37.2 %) public and private schools respectively (χ^2^ = 24.371, P = 0.001). The practice of school health programme was dependent on the age (χ^2^ = 12.53, P = 0.006) and the ethnicity of the respondents (χ^2^ = 6.330, P = 0.042). Using multivariate analysis only one variable (type of school) was found to be a predictor of school health programme. (OR 4.55, CI 1.918–10.79).

**Conclusion:**

The study concludes that the practice of the various components of school health services was poor but better in private primary schools in Nigeria. Routine inspection by teachers was the commonest form of health appraisal. This may suggest that more health personnel need to be employed to cater for the health of the school children in Nigeria and other similar developing countries.

**Electronic supplementary material:**

The online version of this article (doi:10.1186/s13104-016-2006-6) contains supplementary material, which is available to authorized users.

## Background

School health services refer to the health care delivery system that is operational within a school or college. These services aim at promoting and maintaining the health of school children so as to give them a good start in life. In addition, these services seek to enable children benefit optimally from their school learning experience [1, 2]. Globally the number of children reaching school age is estimated to be 1.2 billion children (18 % of the world’s population) and rising [3]. In many homes across the world, children start to attend school from as early as 5–6 months because mothers have to wean early to return to their work place [3]. The purpose of the school health services is to help children at school to achieve the maximum health possible for them to obtain full benefit from their education.

School health services deal with health appraisals, control of communicable diseases, record keeping and supervision of the health of school children and personnel [3, 4]. It is the aspect that concerns itself with the evaluating the health of an individual objectively. Health appraisals afford the school authorities the opportunity to detect signs and symptoms of common diseases as well as signs of emotional disturbances that could impede the learning activities of children [4]. School health services are both preventive and curative services and it helps in providing information to parents and school personnel on the health status of school children [5]. It also provides advisory and counselling services for the school community and parents. It include pre-entry medical screening, routine health screening/examination, school health records, sick bay, first aid and referral services. Other services rendered include health observation (which involves physical inspection of the physiology and behaviours of children), health examinations (screening tests and medical diagnosis) and health records (keeping of records of the health histories of children) [4, 5].

A National study of the school health system in Nigeria by the Federal Ministries of Health and Education revealed that only 14 % of head teachers indicated that pre-enrolment medical examination was mandatory in their schools and 30 % of the students had low body mass index (BMI). It further indicated that 30 % of students have low BMI and the common health conditions that contribute to absenteeism include fever (56 %), headache (43 %), stomach ache (29 %), cough/catarrh (38 %) and malaria (40 %) [4, 5] There is a dearth of school health clinics in Nigeria and where they exist, the services are not comprehensive enough or not organized to meet the needs of the pupils [5]. Studies have shown that primary school children in Nigeria were not provided with basic health examination services and pre-entrance medical examinations thus baseline health information about them was absent. There is also a lack of routine medical examination which would have picked up deviations from normal which make early referrals impossible and children vulnerable to preventable diseases [6, 7].

School health has been described as the neglected component of Primary Health Care in Africa [8, 9]. Since almost every small community has a primary school, in those communities without health centres, it should be possible to use the primary school as a centre for primary health care delivery not just for the pupils but also for the community [10]. A well organized and properly executed school health programme can be used to create safe environment for school children [9]. School health programme can become one of the strategies for promoting primary health care services [11]. All efforts at addressing the school health programme in Nigeria have remained largely at policy level, with minimal implementation. Where implementation has been attempted the emphasis has been on outside rather than within the schools [12–14].

This study was therefore designed to determine the school health services available and its practices in primary schools in Ogun state Nigeria. This has implications in the primary health care of the school children and reduction in incidence of preventable diseases early in life.

## Methods

### Study area

The study was carried out in Ogun state, South West Nigeria. Ogun state was created on February 3rd 1976 out of the defunct Western Nigeria. The state is named after Ogun River which runs right across it from North to South. Ogun state is situated on latitude 7.00° N and longitude 3.35° E in the Greenwich Meridian. It covers a total land area of 16,409.26 km^2^ within the South West region of the country. It is bounded in the north by Oyo and Osun states, in the east by Ondo state, in the west by the Republic of Benin which makes it an access route to the expansive market of the Economic Community of West African States (ECOWAS) and in the south by Lagos state and the Atlantic Ocean. The state Capital Abeokuta, lies about 100 km north of Lagos state, Nigeria’s business Capital [15].

The projected population of the state as at 2012 is 5.1 million. The people of the state belong to the Yoruba ethnic group of South–West Nigeria. The main ethnic groups of the state are Egbas, Ijebus, Remos, Yewas, Eguns and Aworis. Major occupations in the state are farming trading, artisan and white collar jobs. The three major religion of the people are Christianity, Islam and traditional religion. A greater proportion of the state lies in the tropical rain forest zone [15]. The state has twenty (20) Local Government Areas (LGA). Each LGA is headed by an Executive Chairman. It has three (3) Senatorial Districts and is divided into four (4) geo-political zones.

### Study population

The study population consisted of all the head teachers in public and private primary schools in Ogun state and their schools. The Ogun State Universal Basic Education Board (SUBEB) is in charge of primary school education and activities within the state under the Ministry of Education. The state operate a 6-3-3-4 system of education which means 6 years in primary school, 3 years in junior secondary schools, 3 years in senior secondary school and 4 years in the University. There are One thousand, four hundred and forty nine (1449) registered public primary schools and one thousand, six hundred and ninety four (1694) registered private primary schools within the state making a total of 3143 primary schools [6].

The schools have an Administrative Head known as the head teacher and he/she supervises all school activities and the activities of the teaching and non-teaching staff. The head teacher and other staff within the public schools are employed by the State’s Ministry of Education while the private school Heads and Staff are employed by a Proprietor/Proprietress who may also function as the head teacher. All the public schools run the six (6) year programme but some private schools run a five (5) year programme. The private schools usually have an attached Crèche and Nursery Units.

The Zonal Education Office (ZEO) is responsible for compliance and adherence to the Educational standards as specified by the Ministry of Education for all public and private schools within each Zone. In each Local Government, the Local Government Education Authority (LGEA) is directly responsible for the supervision and human resource management of public primary schools. The three (3) Local Government Areas where the study was carried out are Sagamu, Abeokuta South and Ado-Odo/Ota [6].

### Study design

The study design was a comparative cross sectional study that assessed the school health services in public and private primary schools in Ogun state. All fully registered public and private primary schools in the selected LGAs were included in the sampling frame while all unregistered schools were excluded.

### Sample size

A prevalence of 40.4 % of private schools compared to 31.0 % of public schools [16] was used to estimate the sample size using the formula for comparative study proportions between two groups.

$$ N = \frac{{Z_{\alpha } \sqrt {P_{1} (1 - P_{1} )} + Z_{\beta } \sqrt {P_{2} (1 - P_{2} )} }}{{(P_{1} - P_{2} )^{2} }} $$

Thus, a minimum sample size of 153 head teachers is required per group, however a total of *360* participants were recruited into the study.

### Sampling technique

A multi-stage sampling technique was employed.

#### Stage I

A simple random sampling method was used to select three local government areas, one from each of the three senatorial district’s sampling frame which consist of nine from Ogun East, six from Ogun Central and five from Ogun West senatorial districts respectively.

#### Stage II

A simple random sampling method was used to select 60 public and 60 private primary schools from the sampling frame of all the public and private schools in each of the three LGA selected making a total of 360 schools. The head teachers of each schools were recruited into the study.

### Data collection instrument

#### Questionnaire

A self-administered semi-structured questionnaire with open and closed ended questions for the head teachers was designed for the study. It was adapted from that used by Ofovwe and Ofilli [16] in a similar study in 2004. The questionnaire consists of:

Section A: Socio-economic and demographic characteristics such as age, sex, marital status, highest educational qualification and length of time as a head teacher. This section gave insight into the respondents’ socio-economic and demographic background.

Section B: This section contained questions that assessed the head teachers’ knowledge of school health programme.

Section C: This section assessed some of practices of school health programme by the head teachers in their various schools. The section served to augment the main Instrument that was used to assess practice of school health within the schools which was the observational checklist.

#### The observational checklist

was adapted from the school health programme evaluation scale by the Federal Ministry of Education’s sanitary inspection form [17]. The checklist covered all the domains of the school health programme. It was the main Instrument used to evaluate the practice of school health programme.

### Data collection technique

The instruments for data collection: a self-administered semi-structured questionnaire for the head teachers and an observational checklist for the schools were pre-tested in ten (10) public and ten (10) private primary schools in Ibadan North East Local Government and modified as appropriate. Twenty (20) research assistants were recruited and trained in the correct use of the questionnaire (Additional file 1) and the checklist (Additional file 2). Identification tags with pictures were issued to the Research Assistants to facilitate school entry.

School entry was made by approaching the head teacher. Some of the schools randomly recruited into the study were located in ‘hard to reach’ areas with difficult terrains that required very long treks on foot and crossing of rivers in canoes. Once the head teacher give his consent by signing the informed consent form, he/she was given a copy of the questionnaire to fill in the presence of a research assistant who explained grey areas when necessary. Observational checklist was also used to assess practice of school health programme usually in the company of a teacher nominated by the head teacher. Data were collected over a three (3) month period.

### Analysis of results

Quantitative data collected was checked for errors, cleaned, entered and analyzed using the SPSS version 15.0. Data was summarized with proportions and means and presented using frequency tables. The data analysis focused on univariate frequency table and bivariate cross tabulations that identify important relationships between variables. Respondents were categorized into good and poor knowledge status by identifying the correct answer as indicated in the National school health evaluation scale [17]. Practice of school health programmes was described as indicated in the evaluation scale form by the Federal Ministry of Education.

Inferential statistics to test for associations between variables was done using the Chi square test, t test was used to compare the difference between the mean. Logistic regression was then used to estimate predictors of willingness to practice school health programme. Variables that were found to be significant at 0.05 for factors affecting Implementation of school health programme were fed into the Logistic regression model in order to assess the effect of confounding factors. The level of statistical significance was set at 5 %.

### Ethical approval

Ethical approval to conduct the study was obtained from the Ethical Committee of the Olabisi Onabanjo University Teaching Hospital, Sagamu. Official permission was obtained from the office of the permanent secretary, state Ministry of Education and the three (3) Local Government Authorities where the schools were sited. Furthermore, the zonal education officers of Sagamu, Abeokuta South and Ado-Odo/Ota local government areas were also informed.

Written informed consent was obtained from all the participants after study objectives were explained to them. They were assured that participation was voluntary and they would incur no loss if they decided not to participate.

Study participants were assured of strict confidentiality and this was indicated on the questionnaire. Data collected was only used for research purposes and was kept confidential on a password protected computer. Research assistants were also trained not to disclose the information divulged by the respondents during the interview. Anonymity was assured as names or any other personal identifying information was not required from subjects.

## Results

### Socio-dermographic characteristics

All the schools surveyed provide school health services. The mean age of the head teachers in public schools was 53.0 ± 3.6 years while that for the private schools was 37.4 ± 8.0 years. Table 1 shows the socio-dermographic characteristics of the respondents.

**Table 1:** Characteristics of respondents’ socio-demographic variables

Characteristics	Public schools N = 180 (%)	Private schools N = 180 (%)	Total N = 360 (%)	Test statistic value (χ^2^)	p value
Age at last birthday					
21–30	0 (0.0)	30 (16.7)	30 (8.3)		
31–40	2 (1.1)	110 (61.1)	112 (31.1)		
41–50	31 (17.2)	30 (16.7)	61 (16.9)	2.250	0.001
51–60	147 (81.7)	9 (5.0)	156 (43.3)		
≥60	0 (0.0)	1 (0.6)	1 (0.3)		
Sex					
Male	39 (21.7)	51 (28.3)	90 (25.0)	1.809	0.179
Female	141 (78.3)	129 (71.7)	270 (75.0)		
Marital status					
Single	5 (2.8)	31 (17.2)	36 (10.0)		
Married	152 (87.8)	144 (80.0)	302 (83.9)	25.803	0.001
Separated/divorced	3 (1.7)	1 (0.6)	4 (1.1)		
Widowed	14 (7.8)	4 (2.2)	18 (5.0)		
Religion					
Christianity	155 (86.1)	158 (87.8)	313 (86.9)		
Islam	24 (13.3)	20 (11.1)	44 (12.2)	0.966	0.617
Others	1 (0.6)	2 (1.1)	3 (0.8)		
Ethnicity					
Hausa	0 (0.0)	0 (0.0)	0 (0)		
Ibo	18 (10.0)	32 (17.8)	50 (13.8)	5.343	0.069
Yoruba	158 (87.8)	141 (78.3)	299 (83.1)		
Others	4 (2.2)	7 (3.9)	11 (3.1)		
Highest educational qualification					
Masters degree	8 (4.4)	17 (9.4)	25 (6.9)	7.417	0.060
University degree	98 (54.4)	93 (51.7)	191 (53.1)		
Certificate from college of education	69 (38.3)	59 (32.8)	128 (35.6)		
Teacher’s training school certificate	5 (2.8)	11 (6.1)	16 (4.4)		
How long have you been a head teacher (years)					
1–5	93 (51.7)	98 (54.4)	191 (53.1)		
6–10	35 (19.4)	47 (26.1)	82 (22.8)	6.804	0.078
11–15	20 (11.1)	19 (10.6)	39 (10.8)		
>15	32 (17.8)	16 (8.9)	48 (13.3)		

### Knowledge of the respondents about school health services

More than three quarters of the head teachers in both groups could not provide a basic definition of the school health programme. Majority, 166 (92.2 %) of the public and 167 (92.8 %) of the private school head teacher gave a poor definition of SHP (χ^2^ = 2.043, P = 0.360). Furthermore, 164 (91.1 %) of the public and 167 (92.8 %) of the respondents were unable to correctly list the components of the SHP (χ^2^ = 3.327, P = 0.189). Few of the respondents, 40 (22.2 %) of the public and 50 (27.8 %) of the private school head teachers did not know if basic life support is an integral skill needed by the school’s first aider, (χ^2^ = 1.398, P = 0.237). This is shown in Table 2 below.

**Table 2:** Knowledge of respondents’ about school health programme

Knowledge	Public schools N = 180 (%)	Private schools N = 180 (%)	Total N = 360 (%)	Test statistic value (X^2^)	p value
Definition of school health programme					
Poor	166 (92.2)	167 (92.8)	333 (92.5)	2.043	0.360
Fair	12 (6.7)	13 (7.2)	25 (6.9)		
Good	2 (1.1)	0 (0.0)	2 (0.6)		
Knowledge of the components of school health programme					
Poor	164 (91.1)	167 (92.8)	331 (91.9)		
Fair	15 (8.3)	9 (5.0)	24 (6.7)	3.327	0.189
Good	1 (0.6)	4 (2.2)	5 (1.4)		
Knowledge of the impact of medical record keeping in school health programme					
Correct	176 (97.8)	175 (97.2)	351 (97.5)	0.203	0.652
Incorrect	4 (2.2)	5 (2.8)	9 (2.5)		
Effective knowledge of first aid among head teachers					
Correct	140 (77.8)	130 (72.2)	270 (75.0)	1.398	0.237
Incorrect	40 (22.2)	50 (27.8)	90 (25.0)		

### Services available in the schools

There was no health personnel or a trained first aider in 86 (47.8 %) public schools and 110 (61.1 %) private schools. Also, a nurse/midwife was present in only 57 (31.7 %) and 27 (15.0 %) public and private schools respectively (χ^2^ = 17.122, P = 0.002). Periodic medical examination for staff and pupils was carried out in only 13 (7.2 %) public and 31 (17.2 %) private schools. This was a statistically significant finding (χ^2^ = 8.398, P = 0.004).

Essential drugs and materials were totally absent in 66 (36.7 %) of public and 40 (22.2 %) of private schools. (χ^2^ = 9.039, P = 0.003). A sick bay/clinic was present only in 26 (14.4 %) and 67 (37.2 %) public and private schools respectively (χ^2^ = 24.371, P = 0.001). While an ambulance/school bus was present in 5 (2.8 %) of the public schools, 44 (24.4 %) of the private schools had an ambulance or a school bus. (χ^2^ = 35.931, P = 0.001). First aid of any type was unavailable in 33 (18.3 %) public schools and 13 (7.2 %) private schools (χ^2^ = 9.970, P = 0.002). Wash hand basins and stands were present in 32 (17.8 %) and 54 (30.0 %) public and private schools respectively (χ^2^ = 7.394, P = 0.007). This is as shown in Table 3. Routine inspection by teachers was the commonest form of health appraisal done in this study. In about 95 % of the schools, the teacher carried out routine inspection of the pupils. Periodic medical examination was carried out by 17 % of private schools as against 7 % of public schools.

**Table 3:** Practice of school health services in public and private schools

Practice	Public schools N = 180 (%)	Private schools N = 180 (%)	Total N = 360 (%)	Test statistic value (X^2^)	p value
Personnel					
None	86 (47.8)	110 (61.1)	196 (54.4)		
Health assistant/trained first-aider	24 (13.3)	33 (18.3)	57 (15.8)		
Health educator/nutritionist	11 (6.1)	10 (5.6)	21 (5.8)	17.122	0.002
Nurse/midwife	57 (31.7)	27 (15.0)	84 (23.3)		
Doctor	2 (1.1)	0 (0.0)	2 (0.6)		
Health appraisals					
Routine (teacher) inspection					
Yes	168 (93.3)	175 (97.2)	343 (95.3)	3.025	0.082
No	12 (6.7)	5 (2.8)	17 (4.7)		
Screening test for growth defect, handicaps, disabilities					
Yes	14 (7.8)	10 (5.6)	24 (6.7)	0.714	0.398
No	166 (92.2)	170 (94.4)	336 (93.3)		
Periodic medical exams for staff and pupils					
Yes	13 (7.2)	31 (17.2)	44 (12.2)	8.398	0.004
No	167 (92.8)	149 (82.8)	316 (87.8)		
Referrals to health centres/hospitals					
Yes	82 (45.6)	88 (48.9)	170 (47.2)	0.401	0.526
No	98 (54.4)	92 (51.1)	190 (52.8)		
Supervision of health of the handicapped					
Yes	14 (7.8)	7 (3.9)	21 (5.8)	2.478	0.115
No	166 (92.2)	173 (96.1)	339 (94.2)		
Treatment facilities					
First aid box					
Yes	167 (92.8)	162 (90.0)	329 (91.4)	0.882	0.348
No	13 (7.2)	18 (10.0)	31 (8.6)		
Essential drugs and materials					
Yes	114 (63.3)	140 (77.8)	254 (70.6)	9.039	0.003
No	66 (36.7)	40 (22.2)	106 (29.4)		
Sick bay/clinic					
Yes	26 (14.4)	67 (37.2)	93 (25.8)	24.371	0.001
No	154 (85.6)	113 (62.8)	267 (74.2)		
Ambulance/school bus					
Yes	5 (2.8)	44 (24.4)	49 (13.6)	35.931	0.001
No	175 (97.2)	136 (75.6)	311 (86.4)		
Emergency care					
First aid treatment usually given					
Yes	147 (81.7)	167 (92.8)	314 (87.2)	9.970	0.002
No	33 (18.3)	13 (7.2)	46 (12.8)		
Treatment given recorded or referral copy seen					
Yes	39 (21.7)	33 (18.3)	72 (20.0)	0.625	0.429
No	141 (78.3)	147 (81.7)	288 (80.0)		
Notification of parent					
Yes	149 (82.8)	158 (87.8)	307 (85.3)	1.792	0.181
No	31 (17.2)	22 (12.2)	53 (14.7)		
Transport child to nearest health post					
Yes	115 (63.9)	132 (73.3)	247 (68.6)	3.728	0.054
No	65 (36.1)	48 (26.7)	113 (31.4)		
Transport child home afterwards					
Yes	42 (23.3)	34 (18.9)	76 (21.1)	1.067	0.302
No	138 (76.7)	146 (81.1)	284 (78.9)		

### Factors influencing implementation of school health services

The public school head teachers reported lack of infrastructures (51.7 %), lack of funds (42.8 %) and inadequate health personnel (31.1 %) as the three most important challenges that they face in running the SHP. On the other hand, the private school head teachers had listed lack of funds (24.4 %), inadequate health personnel (20.6 %) and friction between parents and the school management (16.1 %) as the three major challenges faced while trying to implement the school health programme.

The study revealed as indicated in Table 4 that the practice of SHP was dependent on the age (χ^2^ = 12.53, P = 0.006) and the ethnicity of the respondents (χ^2^ = 6.330, P = 0.042). It was however not dependent on sex, marital status, religion, highest educational qualification and years of experience (P > 0.05). The practice score of the respondents in public and private schools when compared was dependent on the type of school (χ^2^ = 29.120, P = 0.001).

**Table 4:** Practie of school health programme and socio-demographic variables of respondents

Characteristics of respondents	Poor practice of SHP	Good practice of SHP	Total N = 360 (%)	Test statistic value (X^2^)	p value
Age at last birthday					
21–30	15 (8.0)	15 (8.7)	30 (8.3)	12.53	0.006
31–40	45 (24.1)	67 (38.7)	112 (31.1)		
41–50	30 (16.0)	31 (17.9)	61 (16.9)		
>50	97 (51.9)	60 (34.7)	157 (43.6)		
Sex					
Male	46 (24.6)	44 (25.4)	90 (25.0)	0.33	0.855
Female	141 (75.4)	129 (74.6)	270 (75.0)		
Marital status					
Single	16 (8.6)	20 (11.6)	36 (10.0)	0.971	0.808
Married	159 (85.0)	143 (82.7)	302 (83.9)		
Separated/divorced	2 (1.1)	2 (1.2)	4 (1.1)		
Widowed	10 (5.3)	8 (4.6)	18 (5.0)		
Religion					
Christianity	124(66.3)	104 (60.1)	228 (63.3)	1.485	0.223
Islam	63 (33.7)	69 (39.9)	132 (36.7)		
Ethnicity					
Igbo	23 (12.3)	27 (15.6)	50 (13.9)	6.320	0.042
Yoruba	162 (86.6)	137 (79.2)	299 (83.1)		
Others	2 (1.1)	9 (5.2)	11 (3.1)		
Highest educational qualification					
Masters degree	9 (4.8)	16 (9.2)	25 (6.9)	2.803	0.423
University degree	101 (54.0)	90 (52.0)	191 (53.1)		
College of education	68 (36.4)	60 (34.7)	128 (35.6)		
Teacher’s training school certificate	9 (4.8)	7 (4.0)	16 (4.4)		
Experience of respondents as a head teacher? (years)					
1–5	99 (52.9)	92 (53.2)	191 (53.1)		
6–10	43 (23.0)	39 (22.5)	82 (22.8)	0.267	0.996
11–15	19 (10.2)	20 (11.6)	39 (10.8)		
>15	26 (13.9)	22 (12.7)	48 (13.3)		

Table 5 shows the multiple logistic regression model. Only one variable (type of school) was found to be a predictor of school health programme. (OR 4.551, CI 1.918–10.799).

**Table 5:** Predictors of practice of school health programme (multivariate analysis)

S/N	Variables	Adjusted OR (95 % CI)
1.	Type of school	
	Private	4.551 (1.918–10.799)
	Public	1.00
2.	Age	
	31–40	0.377 (1.121–1.172)
	41–50	0.596 (0.232–1.530)
	>50	0.905 (0.434–1.887)
3.	Ethnicity	
	Igbo	0.214 (0.043–1.055)
	Others	0.264 (0.049–1.423)
	Yoruba	1.00

## Discussion

The importance of a good and functional SHP as a component of Primary Health Care in the overall development of children and the citizenry of a nation cannot be over emphasized. Various studies in the last 20 years or more in Nigeria have indicated poor status of the school health programme [5, 17, 18]. Knowledge of the school health services were generally poor. The generally poor knowledge on school health services has been demonstrated in other previous studies [19–22]. School health services constitute one of the major components of the SHP and deals with the maintenance of the health of the school children. Effective school health services facilitate early detection and diagnosis with prompt intervention in order to prevent mortality and reduce morbidity.

This study showed that almost all of the schools studied did not have the services of a doctor and only one out of every six of the schools in this study had someone trained in first aid. This dearth of health personnel have been reported severally in various studies conducted in Nigeria [23, 24]. This shows that there has not been any improvement in supply of health personnel to school health care in the last 10 years in various parts of Nigeria. The figures from this study and all the other studies above are in sharp contrasts compared with a 1972 study in Ibadan which reported that about two-thirds of the schools had a trained first aider [25]. This may imply a steady deterioration in the SHP within the last four decades as noted by some authors [26, 27]. Every teacher should be trained to be able to administer first aid within the primary school system. However as a minimum requirement three persons trained in first aid should be available at all times in the schools [28].

This study shows that about a quarter of the Schools had a sick bay/clinic while fewer still have any form of school ambulance or bus to convey sick children to hospitals in case of any emergency. Several authors have reported similar findings in their studies [23–25]. Absence of sick bays and school ambulance or bus reflected the poor state of school health services in the schools with the private schools just slightly better.

Routine inspection by teachers was the commonest form of health appraisal done in this study. In about 95 % of the schools, the teacher carried out routine inspection of the pupils. This figure is close to those reported in other studies [19, 29]. Other authors however reported a general absence of health appraisal services [1, 2]. Screening tests for growth defect, handicaps and disabilities were only available in 7 % of the schools in this study. Several studies in Nigeria has reported similar findings [2, 5]. These low figures suggest that most handicaps and disabilities would be discovered much later and at a time when they might have become permanent and irreversible. It has been postulated that a teacher must never be in doubt about the seeing and hearing status of the pupils in his or her class [30].

Periodic medical examination was carried out by few of the respondents’ schools. However higher figures have been reported in previous studies [16, 23] while others studies showed poor medical examinations in schools [19, 31]. Medical officers and other health workers should have schools placed under their watch which they would oversee and help conduct routine medical examination. Pre-entrance medical screening must become an admission requirement into all public and private schools in Ogun state complimented by good record keeping practices at the schools.

On further analysis, the age and ethnicity of the head teacher and the type of school were strong determinants in the practice of school health services. School health services is four times more likely to be implemented in a private when compared to the public school (OR 4.55, CI 1.198–10.79). Private schools have better access to funding because they are also run as a profit-oriented business. Some of the available structures that complement school health programme activities are available because they have to compete with other private schools for pupils. They therefore have a tendency to provide some of the services not because they have an understanding of the requirements of the SHP but as a business model to attract clientele. Public schools on the other hand have to wait for the Government in order to have funds available for all activities. They are usually barred from fund raising activities and when they do the funds are very limited.

The study findings are limited in terms of overall generalization and impact because there may be variation in the availability of resources and political will in the operation of school health services in various LGA in Nigeria and other low income countries. Furthermore, the limitations of a cross-sectional study to explore risk and protective factors are important limitations of this study. Despite these limitations, we believe that our data provide useful information for the assessment of the school health programme in Nigeria and identify factors associated with its practice in Nigeria and other low income countries.

## Conclusion

The study concludes that the practice of the various components of school health services was poor. The health care personnel available in these schools were inadequate but the situation was generally better in the private schools. School health services are four times more likely to be implemented in a private school when compared to the public school. Routine inspection by teachers was the commonest form of health appraisal. This may suggest that more health personnel need to be employed to cater for the health of the school children in Nigeria and other similar developing countries.

Medical officers and other health workers should have schools placed under their watch which they would oversee and help conduct routine medical examination. Pre-entrance medical screening must become an admission requirement into all public and private schools in Nigeria and other countries with similar public health challenges. These inadequacies need to be addressed if health targets such as MDG goals needs to be achieved in Nigeria and other developing countries.

## Additional files

10.1186/s13104-016-2006-6 Questionnaire.
10.1186/s13104-016-2006-6 Check list.
