# Supplementary material for: School health services and its practice among public and private primary schools in Western Nigeria
Source: BMC Res Notes. 2016 Apr 6;9:203. doi: 10.1186/s13104-016-2006-6 (PMC4822242; doi:10.1186/s13104-016-2006-6)
Supplement: Supplementary file 1 — 10.1186/s13104-016-2006-6 Questionnaire. [file 13104_2016_2006_MOESM1_ESM.docx]

**APPENDIX**

**QUESTIONNAIRE**

COMPARATIVE STUDY OF SCHOOL HEALTH PROGRAMME IN PRIVATE AND PUBLIC PRIMARY SCHOOLS IN OGUN STATE.

**SECTION A**

SCHOOL PUBLIC ( ) PRIVATE ( )

1. AGE LAST BIRTHDAY: _____________
2. SEX: MALE ( ) FEMALE ( )
3. MARITAL STATUS: SINGLE ( ) MARRIED ( ) SEPARATED/DIVORCED ( ) WIDOWED ( )
4. RELIGION: CHRISTIANITY ( ) ISLAM ( ) OTHERS ( )
5. ETHNICITY: HAUSA ( ) IBO ( ) YORUBA ( ) OTHERS ( )
6. HIGHEST EDUCATIONAL QUALIFICATION: MASTERS DEGREE ( ) UNIVERSITY DEGREE ( ) CERTIFICATE FROM COLLEGE OF EDUCATION ( ) TEACHERS’ TRAINING SCHOOL CERTIFICATE ( )

1. HOW LONG HAVE YOU BEEN A HEAD TEACHER: 1-5 YEARS ( ) 6-10 YEARS ( ) 11-15 YEARS ( ) >15 YEARS ( )

**SECTION B.**

1. HAVE YOU EVER HEARD ABOUT THE SCHOOL HEALTH PROGRAMME? YES ( ) NO ( )
2. DEFINE SCHOOL HEALTH (SHP) PROGRAMME IN YOUR OWN WORDS.
3. LIST THE COMPONENTS OF SCHOOL HEALTH PROGRAMME THAT YOU KNOW
4. IS SHP A COMPONENT OF PRIMARY HEALTH CARE IN NIGERIA? YES ( ) NO ( ) I DON’T KNOW ( )
5. THE ACTIVITIES OF SHP IS REGULATED BY: LOCAL GOVERNMENT ( ) STATE GOVERNMENT ( ) FEDERAL GOVERNMENT ( ) ALL OF THE ABOVE ( ) NONE OF THE ABOVE ( )
6. SCHOOL NUTRITION SERVICES WILL REDUCE HUNGER AND MALNUTRITION AMONG LEARNERS: TRUE ( ) FALSE ( ) I DON’T KNOW ( )
7. SCHOOL HEALTH SERVICE CENTRE ‘MUST’ AND CAN ONLY BE SITED WITHIN SCHOOL PREMISES: TRUE ( ) FALSE ( ) I DON’T KNOW ( )
8. INSPECTION OF CHILDREN WILL ALSO INCLUDE TOOTH DECAY AND BAD BREATH: TRUE ( ) FALSE ( ) I DON’T KNOW ( )
9. SHP DOES NOT ASSESS CHILDRENS’ IMMUNIZATION STATUS: TRUE ( ) FALSE ( ) I DON’T KNOW
10. PLASTER OF PARIS IS A USUAL CONTENT OF THE FIRST AID BOX: TRUE ( ) FALSE ( ) I DON’T KNOW ( )
11. BASIC LIFE SUPPORT IS AN INTEGRAL SKILL NEEDED BY THE SCHOOL’S FIRST AIDER: TRUE ( ) FALSE ( ) I DON’T KNOW ( )
12. ADVOCACY TO COMMUNITY AND PARENTS IS NOT NECESSARY TO HAVE AN EFFECTIVE SHP: TRUE ( ) FALSE ( ) I DON’T KNOW ( )
13. HEALTH RECORDS OF CHILDREN MUST BE CONSISTENTLY UPDATED PERIODICALLY: TRUE ( ) FALSE ( ) I DON’T KNOW ( )
14. SHP DOES NOT MAKE PROVISION FOR PHYSICALLY AND EMOTIONALLY CHALLENGED CHILDREN: TRUE ( ) FALSE ( ) I DON’T KNOW ( )
15. SCHOOLS SHOULD BE SITED CLOSE TO THE COMMUNITY MARKET AREA TO PROVIDE EASY ACCESS TO TRADER’S CHILDREN: TRUE ( ) FALSE ( ) I DON’T KNOW ( )
16. GOVERNMENT MUST PROVIDE ALL THE FUNDING NEEDED FOR ALL SHP ACTIVITIES: TRUE ( ) FALSE ( ) I DON’T KNOW ( )
17. SHP DOES NOT PLAY A VITAL ROLE IN THE ATTAINMENT OF MILLENIUM DEVELOPMENT GOALS: TRUE ( ) FALSE ( ) I DON’T KNOW ( )
18. SHP IS PART OF WORLD HEALTH ORGANIZATION’S STRATEGY TO PRODUCE ‘HEALTH PROMOTING SCHOOLS’ WORLDWIDE: TRUE ( ) FALSE ( ) I DON’T KNOW ( )
19. THE KEY PERSONNEL/STAFF NEEDED IN THE IMPLEMENTATION OF SHP ACTIVITIES IN SCHOOLS IS __________________

**SECTION C**

1. DO YOU HAVE SCHOOL HEALTH PROGRAMME IN YOUR SCHOOL: YES ( ) NO ( )
2. DO THE PUPILS UNDERGO MEDICAL INSPECTION BEFORE ENTRY INTO THE SCHOOL? YES ( ) NO ( )
3. IF YES, ARE THERE RECORDS AVAILABLE? YES ( ) NO ( )
4. IF NO, WHY IS IT NOT DONE? _____________________________
5. DO YOU PERFORM ROUTINE MEDICAL INSPECTION ON THE PUPILS? YES ( ) NO ( )
6. IF YES, ARE RECORDS AVAILABLE? YES ( ) NO ( )
7. IF NO, WHY IS IT NOT DONE? _______________________________
8. WHO CONDUCTS THE MEDICAL INSPECTION? DOCTOR ( ) NURSE ( ) CHEW ( ) HEALTH ATTENDANT ( ) TEACHER ( )
9. DO YOU HAVE A FIRST AID BOX? YES ( ) NO ( )
10. DO YOU HAVE A SCHOOL CLINIC OR SICK BAY? YES ( ) NO ( )
11. IF YES, IS THERE A HEALTH WORKER IN THE SICK BAY? YES ( ) NO ( )
12. IS THERE A SCHOOL MEAL POLICY IN THE SCHOOL? YES ( ) NO ( )
13. WHAT ARE THE SOURCES OF SCHOOL MEAL? HOME ( ) MOBILE FOOD VENDORS ( ) PERMANENT FOOD VENDORS ( )
14. ARE FOOD HANDLERS SCREENED FOR DISEASES? YES ( ) NO ( )
15. WHAT DISEASES ARE THEY SCREENED FOR? ____________________
16. HOW MANY HOURS IN A WEEK ARE SCHEDULED FOR SKILLS BASED HEALTH EDUCATION AND PROMOTION? NONE ( ) 1 ( ) 2 ( ) 3 ( ) 4 AND ABOVE ( )
17. ARE PUPILS ENGAGED IN PHYSICAL EDUCATION? YES ( ) NO ( )
18. IS THERE COUNSELLING AND PSYCHOLOGICAL SERVICES THAT CATER FOR MEDICAL CONDITIONS DISCOVERED IN CHILDREN? YES ( ) NO ( )
19. IS THERE A FUNCTIONAL SCHOOL HEALTH COMMITTEE? YES ( ) NO ( )
20. IS THERE A FUNCTIONAL PARENTS- TEACHERS ASSOCIATION OR SCHOOL BASED MANAGEMENT COMMITTEE? YES ( ) NO ( )

**SECTION D**

1. ARE YOU AWARE OF THE ‘NATIONAL SCHOOL HEALTH POLICY IN NIGERIA’? YES ( ) NO ( )

If No, go to question 50.

1. HAVE YOU SEEN A COPY OF IT? YES ( ) NO ( )

If No, go to question 50.

1. DO YOU HAVE A COPY OF IT IN THE SCHOOL? YES ( ) NO ( )
2. ARE YOU AWARE OF THE ‘IMPLEMENTATION GUIDELINES ON NATIONAL SCHOOL HEALTH PROGRAMME’? YES ( ) NO ( )

If No, go to question 53

1. HAVE YOU SEEN A COPY OF IT? YES ( ) NO ( )

If No, go to question 53

1. DO YOU HAVE A COPY OF IT IN THE SCHOOL? YES ( ) NO ( )
2. LIST THE THREE MOST IMPORTANT CHALLENGES THAT YOU FACE IN RUNNING THE SCHOOL HEALTH PROGRAMME IN YOUR SCHOOL.
