# Supplementary material for: School health services and its practice among public and private primary schools in Western Nigeria
Source: BMC Res Notes. 2016 Apr 6;9:203. doi: 10.1186/s13104-016-2006-6 (PMC4822242; doi:10.1186/s13104-016-2006-6)
Supplement: Supplementary file 2 — 10.1186/s13104-016-2006-6 Check list. [file 13104_2016_2006_MOESM2_ESM.docx]

**CHECK LIST**

**TYPE OF SCHOOL** PRIVATE [ ] PUBLIC [ ]

**AGE/DATE ESTABLISHED**

**SCHOOL POPULATION**

1. TOTAL NO OF CHILDREN : MALE FEMALE
2. TEACHING STAFF:
3. NON-TEACHING STAFF:

|  | **SCORES OBTAINABLE** | | **SCORES OBTAINED** | |
| --- | --- | --- | --- | --- |
| **PERSONNEL** |  | |  | |
| NONE | 0 | |  | |
| HEALTH ASSISTANT/TRAINED FIRST-AIDER | 1 | |  | |
| HEALTH EDUCATOR/NUTRITIONIST | 2 | |  | |
| NURSE/MIDWIFE | 3 | |  | |
| DOCTOR | 4 | |  | |
| **HEALTH APPRAISALS** |  | |  | |
| ROUTINE INSPECTION (TEACHER OBSERVATION) | 1 | |  | |
| SCREENING TESTS FOR GROWTH DEFECT, HANDICAPS, DISABILITIES | 1 | |  | |
| PERIODIC MEDICAL EXAMS FOR STAFF & PUPILS | 1 | |  | |
| REFERRALS TO HEALTH CENTRES/HOSPITALS | 1 | |  | |
| SUPERVISION OF HEALTH OF THE HANDICAPPED | 1 | |  | |
| **TREATMENT FACILITIES** |  | |  | |
| FIRST AID BOX | 1 | |  | |
| ESSENTIAL DRUGS & MATERIALS | 1 | |  | |
| SICK BAY/CLINIC | 1 | |  | |
| AMBULANCE/SCHOOL BUS | 1 | |  | |
| TELEPHONE SERVICES | 1 | |  | |
| **EMERGENCY CARE** |  | |  | |
| FIRST AID TREATMENT USUALLY GIVEN | 1 | |  | |
| TREATMENT GIVEN RECORDED OR REFERRAL COPY SEEN | 1 | |  | |
| NOFICATION OF PARENT | 1 | |  | |
| TRANSPORT CHILD TO NEAREST HEALTH POST | 1 | |  | |
| TRANSPORT CHILD HOME AFTERWARDS | 1 | |  | |
| **DISEASE CONTROL** |  | |  | |
| NO ACTIVITY | 0 | |  | |
| HEALTH TALKS | 1 | |  | |
| SEND CHILD HOME | 1 | |  | |
| ISOLATE/QUARANTINE CHILD | 1 | |  | |
| INITIATE IMMUNIZATION WHERE POSSIBLE | 1 | |  | |
| **HEALTH RECORDS** |  | |  | |
| NO RECORDS AVAILABLE | 0 | |  | |
| AVAILABLE BUT NOT CUMMULATIVE | 1 | |  | |
| CUMMULATIVE BUT NOT TRANSFERABLE | 2 | |  | |
| CUMMULATIVE AND TRANSFERABLE | 3 | |  | |
| **NUTRITIONAL SERVICES** |  | |  | |
| SCHOOL MEALS AVAILABLE | 3 | |  | |
| SCREENING OF VENDORS/FOOD HANDLERS | 3 | |  | |
| TRAINING & CERTIFICATION OF FOOD HANDLERS/ VENDORS | 2 | |  | |
| CLEANLINESS OF FOOD AREA | GOOD – 2, FAIR – 1, POOR – 0 | |  | |
| NUTRITIONAL SUPPLEMENTS | 1 | |  | |
| **HEALTH INSTRUCTION** |  | |  | |
| ONE PERIOD/WEEK | 1 | |  | |
| TWO PERIODS PER WEEK | 3 | |  | |
| THREE PERIODS PER WEEK | 5 | |  | |
| **WATER SUPPLY** |  | |  | |
| PIPE BORNE | 4 | |  | |
| BORE HOLE/MONO PUMP | 3 | |  | |
| WELLS | 2 | |  | |
| SURFACE WATER | 1 | |  | |
| WITHIN THE SCHOOL | 3 | |  | |
| <200 METERS OUTSIDE SCHOOL | 2 | |  | |
| >200 METERS OUTSIDE SCHOOL | 1 | |  | |
| **REFUSE DISPOSAL** |  | |  | |
| INCINERATION | 4 | |  | |
| CONTROLED TIPPING | 3 | |  | |
| COMPOSTING | 2 | |  | |
| OPEN DUMPING/BURNING | 1 | |  | |
| **SEWAGE DISPOSAL** |  | |  | |
| WATER CLOSET/SEPTIC TANK | 3 | |  | |
| PIT/TRENCH | 2 | |  | |
| BUCKET | 1 | |  | |
| SURFACE (BUSH/WATER) | 0 | |  | |
| GENDER DIFFERENTIATED TOILETS | 3 | |  | |
| TOILET ROLLS AVAILABLE | 1 | |  | |
| SOAP FOR HANDWASH AVAILABLE | 2 | |  | |
| STATE OF TOILET AND TOILET AREA | GOOD- 5, FAIR- 3, POOR- 1 | |  | |
| **TOILET-PUPIL RATIO** |  | |  | |
| 1:<30 | 5 | |  | |
| 1: 31-45 | 4 | |  | |
| 1: 46-60 | 3 | |  | |
| 1: 61-90 | 2 | |  | |
| 1:>90 | 1 | |  | |
| NONE | 0 | |  | |
| **BUILDING** |  | |  | |
| STRONG WALS & ROOF | 4 | |  | |
| STRONG WALLS WITH MINOR CRACKS | 3 | |  | |
| OLD WALLS, LEAKING ROOFS | 2 | |  | |
| DILAPIDATED | 0 | |  | |
| **FIRE PROTECTION** |  | |  | |
| ALL BUILDINGS WITH FIRE RESISTANT MATERIAL | 3 | |  | |
| SOME PREFAB BUILDING | 2 | |  | |
| ALL PREFAB BUILDINGS | 1 | |  | |
| **FLOOR** |  | |  | |
| FLAT, NON-GLOSSY | 3 | |  | |
| FLAT GLOSSY | 2 | |  | |
| WORN OFF, BROKEN & DUSTY | 1 | |  | |
| SANDY | 0 | |  | |
| **VENTILATION** |  | |  | |
| ADEQUATE | 2 | |  | |
| NOT ADEQUATE | 1 | |  | |
| CONTROLLABLE | 2 | |  | |
| NOT CONTROLLABLE | 1 | |  | |
| **LIGHTING** |  | |  | |
| LIGHTING | GOOD – 2, POOR -1, SUPPLEMENTARY LIGHT +1 | |  | |
| **INSULATION** |  | |  | |
| INSULATION | PROPERLY CEILED- 2, PARTIALLY CEILED- 1, NO CEILING- 0 | |  | |
| **SITTING COMFORT** |  | |  | |
| PUPILS | 100% SEATED- 2, <100% SEATED- 1, NONE- 0 | |  | |
| TEACHERS | 100% SEATED- 2, <100% SEATED- 1, NONE- 0 | |  | |
| **SAFETY MEASURES** |  | |  | |
| SCHOOL FENCE | 1 | |  | |
| FIRE EXTINGUISHER | 1 | |  | |
| FIRE ALARM | 1 | |  | |
| SAFETY PATROL TEAM | 1 | |  | |
| **NUISANCE & HAZARDS** |  | |  | |
| NOISE POLLUTION | ABSENCE – 5, PRESENCE IN ANY FORM – 0 | |  | |
| FLOODING/OPEN DRAINAGES | ABSENCE- 3, PRESENCE IN ANY FORM- 0 | |  | |
| **HEALTHFUL LIVING** | |  | |  |
| ADEQUATE EMOTIONAL CLIMATE | | 1 | |  |
| SHOES WORN COMPULSORILY BY STAFF & PUPILS | | 1 | |  |
| SPORTS FIELD AVAILABLE | | 1 | |  |
| OTHER SPORTS FACILITIES | | 1 | |  |
| WASH HAND BASINS & STANDS IN CLASS | | 1 | |  |
| DUST BINS & WASTE PAPER BASKETS AVAILABLE | | 1 | |  |
| PRESENCE OF HEALTH BASED IEC MATERIALS | | 2.5 | |  |
| PRESENCE OF HEALTH BASED CLUBS/SOCIETIES/ACTIVITIES | | 2.5 | |  |
| **POLICY MATERIALS** | |  | |  |
| COPY OF NATIONAL POLICY ON SHP IN SCHOOL | | 5 | |  |
| COPY OF IMPLEMENTATION GUIDELINES IN SCHOOL | | 5 | |  |
| **TOTAL OBTAINABLE** | | **120** | |  |
